# Book Reviews

**Published:** 1893-06

**Authors:** 


### *BOOK REVIEWS.*

---

Modern Gynecology. By Chas. H. Bushong, M. D., Attending Gynecologist to the Demilt Dispensary, New York, and Assistant to the Vanderbilt Clinic, College of Physicians and Surgeons. E. B. Treat, 5 Cooper Union, New York.

The object of this book (says the author) is to place before the physician a clear common sense statement of the symptoms of the various diseases of the female sexual organs; to indicate in detail the methods of treatment that can be applied by him, and also to indicate in brief the methods requiring the aid of a specially trained consultant of larger experience. The book has been written for the general practitioner, not the specialist; and those physicians who are called upon to treat the diseases of women in a general way will find this work a very serviceable guide. While the author is generally conservative, he indicates with much clearness the conditions which demand radical interference.

---

Chronic Disorders of Digestive Tube. By W. W. Vanvalzah, M. D., formerly Demonstrator of Clinical Medicine, Jefferson Medical College, New York. J. H. Vail & Co.

This volume of one hundred and fifty pages is a reprint of several articles which the author contributed to the journals last year. Different chapters describe Chronic Disorders of Gastric Digestion, Intestinal Indigestion, Causation and Treatment of Chronic Diarrhœa, Curative Treatment of Habitual Constipation, etc. An appendix discusses the treatment of Functional and Catarrhal diseases of the Stomach and Bowels, and the Nature and Preventive Treatment of Seasickness. As to the prevention of seasickness the author recommends the adoption of means to secure healthy nutrition and a healthy operation of the intestinal functions for several days before the voyage, and rest in bed for the first forty-eight hours of the trip. The bromides, atropia, strychnine, etc., are often indicated during the attack.

PSYCHOPATHIA SEXUALIS. By Dr. R. von Krafft-Ebing, Professor of Psychiatry, University of Vienna. Translated from the seventh German edition by Dr. Chas. G. Chaddock, M. D., Professor of Nervous and Mental Diseases, Marion-Sims College, St. Louis, Mo. F. A. Davis Company, Philadelphia.

This work is unique. Few authors have gone into this territory, and none probably with the completeness of the present. The subject here presented is a disgusting one, and one almost questions the wisdom of recording the filthy details of sexual peversion. But the pathology of the sexual instinct is now a matter of frequent medico-legal interest and importance, and hence the value of a work in which this subject is ably discussed in the light of scientfic medicine. The book has been prepared for physicians and lawyers; these it will enable to reach something of an understanding of the "remarkable cases" which they now and then see. The author has had a rich experience from which to make his observations, and about two hundred cases are reported. In the first two sections the author discusses the physiology and psychology of the sexual life. Under pathology are described sadism, perversion, masochism, fetichism, contrary sexual instinct, urnings, etc., with reports of many cases. Under treatment the author attaches much importance to hypnotic suggestion, and relates cases thus cured. The last section of the volume is about the legal aspects of pathological sexuality.

Upon reading this volume one easily sees that it is the work of a master. It is for the most part a truthful description of what the author has himself seen with scientific deductions therefrom. It fills a hiatus in medical and legal literature, and to the lawyers and doctors alike it is a work of immense value.

---

A TEXT-BOOK OF THE THEORY AND PRACTICE OF MEDICINE BY AMERICAN TEACHERS. Edited by William Pepper, M. D., LL. D., Professor of the Practice of Medicine and of Clinical Medicine in the University of Pennsylvania. In two volumes. Vol. I. Philadelphia, W. B. Saunders, 913 Walnut St.

The first volume of this work represents the work of six American teachers of medicine who can speak with authority on the subjects which they describe. The excellent section on Hygiene has been prepared by Dr. J. S. Billings, whose professional life has been a labor in this special direction. Dr. Pepper describes the fevers.

The Exanthemata are described by Dr. Jas. T. Whittaker, of Cincinnati. The diseases of the Brain and Nervous System are exhaustively discussed by Drs. H. C. Wood of Philadelphia, and William Osler of Baltimore. Dr. Gilwan Thompson writes the Sections on Syphilis, Erysipelas, Cholera, Malarial Fevers, Diphtheria and Yellow Fever.

These diseases are herein described and discussed with a fullness that does not necessarily obtain in any practice, the work of a single author. No one author can now do justice to the general subject of medicine, and therefore the necessity of composite works is being recognized in nearly every department of medicine and surgery. Between the single volume work on practice which does not contain enough, and the exhaustive and bulky treatise of many volumes containing more than the busy physician generally takes time to read, there is an intermedium which is well represented, we think, by the present work. It has been written within the last few months (as the chapter on cholera indicates), and we may therefore assume that it is more nearly a modern practice than any other. It appears to have been written especially to meet the needs of the practitioner, and we think it justly entitled to the first position as a practical and standard work of reference.

---

### LIPPINCOTT'S MAGAZINE FOR JUNE, 1893.

The complete novel in the June number of *Lippincott's* is "The Translation of a Savage," by Gilbert Parker, author of "The Chief Factor," "Pierre and his People," "Mrs. Falchion," etc. It has an unusual subject, and tells how an Englishman of family and wealth married an Indian girl of Hudson's Bay and took her home, with results naturally mixed, but better than might have been expected.

The fourth in the series of *Lippincott's* notable stories—"The Philosophers," by Geraldine Bonner—deals with an extraordinary wedding, in which the men concerned were philosophers indeed. It is illustrated. "Ambition," a play in one act, by Johanna Staats, has a double love-story.

The Athletic Series is continued in an illustrated article on "Amateur Rowing," by John F. Huneker. In the Journalistic Series, Theodore Stanton descants on "The Foreign Correspondent."

John Burroughs gives "A Glance into Walt Whitman," and Frank A. Burr tells "How Men Write," with portraits of Captain King, J. G. Blaine, Julian Hawthorne, Eugene Field, Joel Chandler Harris, J. W. Riley, Bill Nye and Walt Whitman.

W S. Walsh supplies anecdotes illustrating the methods, now more honored in the breach than in the observance, of "The Practical Jester." Alfred Stoddart, in "An Actor's Art," contributes a brief study of Edward S. Willard. "A Colonial Vista," by F. H. W., is a notice of Miss Wharton's "Through Colonial Doorways." "When Doctors Differ," by F. M. B., is a comment on a recent deliverance of Mr. F. Marion Crawford.

M. Crofton, in "Men of the Day," offers pen-pictures of Ambassador Bayard, Millionaire Mackay, Composer Verdi, and Editor Burnand.

The poetry of the number is unusually full, containing lyrics by Graham R. Tomson, the late Philip Bourke Marston, Lorimer Stoddard, Bliss Carman, and Harrison S. Morris, besides quatrains by Frank Dempster Sherman, Clinton Scollard and Joel Benton.

---

WHAT would you do if assured by scientific men that the world would come to an end within the next twelve weeks? The long promised novel of Can lle Flammarion, "Omega: The last Days of the World," proves to be of thrilling interest. It is the conception of one of the world's most distinguished astronomers, worked out within the bounds of scientific possibility. While educating the reader in the most modern phase of science, it is as full of interesting surprises as the Arabian Nights Entertainment. The most interesting part of this wonderful novel is found in his description of the trepidation and expectation into which the people of the world are thrown. Imagine the condition of the stock exchange with a fact of such import staring them in the face. The opening chapters will be found in the April number of *The Cosmopolitan* magazine.

Probably no novel has ever been presented in an American magazine with such illustrations as accompany Flammarion's "Omega," which commences in the April *Cosmopolitan*. In the list of illustrations are to be found the names of Jean Paul Laurens, Rochegrosse, Chovin, Vogel, O. Saunier, Gerardin and Meaulle.

